# miR-206 as a prognostic and sensitivity biomarker for platinum chemotherapy in epithelial ovarian cancer

**DOI:** 10.1186/s12935-020-01623-y

**Published:** 2020-11-03

**Authors:** Xiaotang Yu, Xinchen Zhang, Guang Wang, Bo Wang, Yanfang Ding, Jinyao Zhao, Hanlin Liu, Shiying Cui

**Affiliations:** 1 Department of Pathology and Forensic Medicine, College of Basic Medicine Sciences, Dalian Medical University, No 9 LvShun South Road-W, Dalian, 116044 China; 2 Department of Histology and Embryology, Dalian Medical University, College of Basic Medicine Sciences, No 9 LvShun South Road-W, Dalian, 116044 China; 3 Cancer Stem Cell Institute, Dalian Medical University, No 9 LvShun South Road-W, Dalian, 116044 China

**Keywords:** Epithelial ovarian cancer, Platinum, Chemoresistance, miR-206

## Abstract

**Background:**

Drug resistance is a major obstacle to successful chemotherapy for epithelial ovarian cancer (EOC). We found a subset of miRNAs associated with the response to first-line platinum-based chemotherapy in EOC by microarray, and miR-206 was one of the most significant miRNAs. The purposes of this study were to evaluate the prognostic and platinum-resistance predictive value of miR-206 in EOC patients and to investigate the functional roles of miR-206 in regulating the platinum resistance of EOC and the underlying mechanism.

**Methods:**

MiRNA expression profiling in EOC specimens was performed using a TaqMan miRNA array. miR-206 expression was confirmed by quantitative real-time PCR (qRT-PCR) analysis. Overexpression of miR-206 in EOC cell lines was achieved by the stable transfection of a recombinant plasmid. In vitro assays of cisplatin cytotoxicity, cell cycle distribution, apoptosis, transwell invasion and cell scratching were employed. Connexin 43 (Cx43) expression was detected by Western blotting. Murine xenograft models were used to determine the effects of miR-206 on platinum resistance in vivo.

**Results:**

miR-206 expression was increased in primary platinum-resistant EOC. High miR-206 expression was related to poor prognosis in EOC patients who received platinum-based chemotherapy and predicted chemoresistance to platinum treatment. Overexpression of miR-206 in cisplatin-sensitive EOC cell lines significantly increased cell viability, migration and invasion in the presence of cisplatin and decreased cisplatin-induced apoptosis. Cx43, a target gene of miR-206, was negatively regulated by miR-206 in EOC cell lines and significantly related to better prognosis in patients who received platinum-based chemotherapy (KmPlot). miR-206 had high expression and Cx43 had low expression in platinum-sensitive EOC cell lines compared with resistant ones. In vivo murine xenograft models showed that miR-206 profoundly promoted the chemoresistance of EOC to cisplatin treatment.

**Conclusion:**

miR-206 was highly expressed in primary platinum-resistant EOCs and functionally promoted platinum resistance in part by downregulating Cx43 expression, thereby providing a useful biomarker for prognostic and platinum-resistance prediction.

## Introduction

Ovarian cancer is one of the four most common malignant tumors and the most lethal gynecologic malignancy, with an associated annual mortality rate of 152,000 [1]. Epithelial ovarian cancer (EOC), which accounts for approximately 90% of ovarian cancer, has a poor prognosis due to late diagnosis and a high incidence of chemoresistance [2]. More than 70% of patients with ovarian cancer are diagnosed at an advanced stage (FIGO III and FIGO IV). The 5-year survival in such patients is less than 30% because of a lack of effective biomarkers for basic standard chemotherapy, prognosis, and personalized treatment [3]. Adjuvant chemotherapy drug resistance is a major cause of decreased overall survival in patients with advanced ovarian cancer. Platinum-based adjuvant chemotherapy is currently considered the standard of care for patients with advanced stage ovarian cancer following primary surgical cytoreduction, especially for serous ovarian cancer (OSC) [4]. Although most patients initially experience a clinical complete response (CR) to adjuvant chemotherapy, a minority (30–40%) will have an incomplete response (IR) or progressive disease despite therapy [4]. Because of the lack of efficient biomarkers to predict chemoresistance, patients with such “platinum-resistant” tumors often receive multiple cycles of platinum-based chemotherapy without clinical benefit, lose the chance of the timely initiation of treatment with active agents, and often have a poor prognosis.

MicroRNAs (miRNAs) are a class of short, single-stranded, noncoding RNAs that are involved in the posttranscriptional regulation of genes through messenger RNA (mRNA) silencing [5]. A single miRNA targets and changes the expression of many genes. Using high-throughput technology, such as microarrays and quantitative RT-PCR for validation, many studies have found associations between miRNA expression levels and tumor type, biological behaviour, grade, response to treatment and prognosis [6]. These studies indicate the vital roles of miRNAs in neoplasia and the potential for miRNAs to serve as biomarkers of disease state and prognosis and predictors of drug resistance [7]. The mechanisms underlying platinum chemotherapy resistance are not fully understood, and no definite biomarkers that predict the response to platinum drugs have been found. The expression signatures of local or systemically circulating miRNAs that are underexpressed (tumor suppressors) or highly expressed (oncogenes) can serve as biomarkers for discriminating tumor origins or subtypes and directing chemotherapy [7, 8].

In the present study, by comparing the miRNA microarray profiles of cancer tissues from EOC patients who showed CR or IR to primary platinum-based chemotherapy, we found a subset of miRNAs that were differentially expressed in the CR and IR groups. Among these, miR-206 was one of the most significantly increased miRNAs in IR patients, and high miR-206 expression was strongly associated with poor patient prognosis. In vitro and in vivo studies confirmed that miR-206 was involved in the EOC response to cisplatin treatment. Our results suggested that miR-206 can be used as a biomarker to predict sensitivity to platinum-based chemotherapy and survival in ovarian cancer patients.

## Materials and methods

### Patients and specimens

Patients who were surgically treated for ovarian cancer between 2004 and 2010 at the Obstetrics and Gynecology Hospital of Dalian (Liaoning, China) were recruited into this study. All participants signed a consent form prior to the surgical procedure and the investigations. The present study was approved by the Institutional Review Board of the Ministry of Science and Technology of China, the Human Resource Management Office (Beijing, China) and the ethics committee of Dalian Medical University (Dalian, China). Clinicopathological data were collected, including subtype, age, FIGO stage, grade, status of lymphatic metastasis, survival and response to therapy. The therapeutic response was evaluated as previously reported [2]. In brief, according to the response to first-line platinum-based chemotherapy, the patients were classified into two groups. A complete response (CR) was defined as the complete disappearance of all measurable and assessable disease or, in the absence of measurable lesions, a normalization of the CA-125 (< 30 U/mL) level following 3 cycles of adjuvant therapy. An incomplete response (IR) was classified as only a partial response, no response, or disease progression during primary therapy or any increase in the CA-125 level from baseline at study entry. The CA-125 response criteria were based on established guidelines [9, 10].

All patients underwent primary surgical cytoreduction followed by adjuvant treatment with platinum-based chemotherapy. The tumor specimens comprised primary ovarian cancer obtained from surgery prior to chemotherapy collected for the experiments. The slides for each case were evaluated by two expert pathologists. Only specimens containing > 70% tumor tissue were used for subsequent experiments. Seventeen EOC ovarian cancer tissues (formalin fixed paraffin embedded blocks) were collected for the miRNA array assay. Among these, 9 patients showed CR, and 8 showed IR after primary platinum-based therapy following surgery (Table 1). In the validation experiment, a separate cohort of patients was recruited from the Obstetrics and Gynecology Hospital of Dalian. Among these patients, 20 showed CR, and 19 showed IR after primary platinum-based therapy (Table 1).

**Table 1:** Clinicopathologic characteristics of epithelial ovarian cancer patients

Parameters	Microarray		Validation cohort (qRT-PCR)	
	CR	IR	CR	IR
Number	9	8	20	19
Age (Mean ± SEM)	49.4	48.7	51.3	50.46
FIGO stage				
I	1	1	1	1
II		2	2	1
III	8	4	17	15
IV		1	0	2
Subtype				
Low grade serous carcinoma	1	1	1	3
High grade serous carcinoma	8	5	19	16
Clear cell carcinoma	0	1		
Endometrioid carcinoma	0	1		
Mean serum CA125, m/ml				
Before platinum	952.1	1872.2	1023.1	1764.2
After platinum	17.2	343.1	14.3	294.3
Mortality rate (%)			48.1%	75%

CR, Clinical complete responders; IR, Clinical incomplete responders

### Cell culture

The cisplatin-sensitive cell line OV2008 was derived from a patient with ovarian serous cystadenocarcinoma who did not undergo prior chemotherapy, and its resistant variant, C13, originated from in vitro cisplatin challenges of OV2008 cells [11]. Another cisplatin-sensitive cell line, A2780, which was established from tumor tissue from an untreated patient and has been implicated as ovarian endometroid adenocarcinoma [12], and its corresponding cisplatin-resistant variant A2780/cis were also used. These cells were acquired as a gift from Professor Chun Peng (Department of Biology, York University, Toronto, Canada). These cell lines were maintained in RPMI-1640 (1x) medium supplemented with 10% fetal bovine serum (FBS) (Gibco, Thermo Fisher Scientific Inc., Waltham, MA, USA) and 1% PSA, sodium bicarbonate (24 mM) and HEPES (10 mM). All cell cultures were maintained at 37 °C in a humidified atmosphere with 5% CO_2_.

### RNA extraction

Total RNA was extracted from the FFPE EOC tissue samples using the Ambion mirVana microRNA Isolation Kit (Ambion Life Technologies, Austin, TX, USA). In brief, tissue deparaffinization was performed by gradient alcohol and xylene followed by protease digestion to remove the proteins covalently bound to RNA. The nucleic acid was isolated by an ethanol mixture and captured on a glass-fiber filter. The final nucleic acid was purified by high-ethanol washing and elution. The quantity and quality of the total RNA was verified with the Agilent RNA 6000 Nano Kit and the BioanalyzerTM (Agilent Technologies, Palo Alto, CA, USA).

### MiRNA microarray and data analysis

The miRNA microarray was performed at the Shannon McCormack Advanced Molecular Diagnostics Laboratory Research Services, Dana Farber Cancer Institute, Harvard Clinic and Translational Science Center (Boston, MA, USA). A microarray platform optimized for the analysis of a panel of 768 human miRNAs (TaqMan® Array Human MicroRNA Card Set v2.0; Thermo Fisher Scientific Inc., Waltham, MA, USA) was used to analyze and compare the patterns of miRNA expression between CR and IR to platinum-based chemotherapy in EOC patients. Individual real-time quantitative polymerase chain reaction assays were formatted into a TaqMan low-density array (TLDA; Applied Biosystems). The normalized microarray data were managed and analyzed using StatMiner version 3.0 (Integromics™, Granada, Spain).

### Quantitative real-time PCR (qRT-PCR)

The miR-206 expression pattern was determined by qRT-PCR using a commercial TaqMan™ MicroRNA Reverse Transcription Kit specific to human miR-206 (Applied Biosystems, Thermo Fisher Scientific, USA). In brief, miR-206 was generated from 220 to 300 ng of the total RNA in a single-step reaction by performing reverse transcription according to the manufacturer’s instructions. PCR amplification was performed in a 96-well optical plate at 95 °C for 10 min, followed by 40 cycles of 95 °C for 15 s and 60 °C for 60 s, using U6 as a housekeeping gene control. The experiments were run in triplicate for each case to allow for technical variability. Real-time PCR was performed on an Agilent Technologies Stratagern MX3000P. The data were analyzed with Mxpro software.

### Stable transfection of miR-206 by plasmid

The recombinant plasmid p-EGP-miR-206, which contained pre-miR-206 cloned into the pEGP-miR vector (5.0 kb), and its vector control pEGP-miR Null Control Vector were gifts from Dr Jin (Feinberg Cardiovascular and Renal Research Institute, Feinberg School of Medicine, Northwestern University, Chicago). The p-EGP-miR-206 plasmids were transformed into competent E. coli DH5α cells (Takara Bio Inc. Dalian, China), and the bacterial strains were amplified in 1 × LB supplemented with ampicillin. Then, plasmids were isolated by the E.Z.N.A. Endo-free Plasmid Mini Kit (Omega Bio-tek, Norcross, GA, USA). The p-EGP-miR-206 and pEGP-miR Null Control Vector plasmids were transfected into the A2780s and OV2008 cell lines by using Lipofectamine™ 2000 reagent (Thermo Scientific, Carlsbad, CA, USA) according to the manufacturer’s instructions. Four days after transfection, the cells were selected by challenging the cells with the proper concentration of puromycin (2 µg/mL) to generate positive clones. Then, the puromycin-resistant clones were selected and cultured. At 4 weeks after cloning, we finally obtained cell lines with stable expression of pEGP-miR-206 (A2780s-206, OV2008-206) and pEGP-miR (A2780s-con, OV2008-con).

### MTS cell viability assay

Cell survival was determined using the Cell Titer AQueous Non-Radioactive Cell Proliferation Assay Kit (Cat# P9625; Promega Co., USA). In brief, cells were cultured in 96-well plates at a density of 1000 cells/well and then treated with 0, 5, 10, 15, 20, 25, 30, 40, 50, and 60 µM cisplatin for 48 h for A2780s cells or 0, 2.5, 5, 7.5, 10, 15, 20, 30, 40, and 50 µM cisplatin for 48 h for OV2008 cells. Twenty microliters of MTS/PMS solution composed of a novel tetrazolium compound [3-(4,5-dimethylthiazol-2-yl)5-(3-carboxymethoxyphenyl)-2-(4-sulfophenyl)-htetrazolium, inner salt; MTS] and an electron coupling reagent (phenazine methosulfate; PMS) at a ratio of 20:1 and 80 μL of complete culture solution were added to one 96-well plate and incubated at 37 °C in a humidified 5% CO_2_ atmosphere for 3 h. The absorbance at 490 nm was measured using an ELISA plate reader (PowerWavex 340, Bio-Tek Instruments Inc., Winooski, VT, USA). Each point represents the mean ± S.D. of triplicates. Responses to drug treatment were assessed by standardizing the treatment groups to the untreated controls. The IC50 was calculated by the Bliss method.

### Cellular apoptosis assay with flow cytometry

For the cellular apoptosis assay with flow cytometry, Annexin V/propidium iodide (PI) staining was performed using the Annexin V-FITC Apoptosis Detection Kit (KGA106; KeyGen Biotech, Nanjing, China). Ovarian cancer cells were initially seeded at a concentration of 5 × 10^5^ cells/mL in 6-well plates and incubated for 24 h. OV2008 cells and C13 cells were then treated with 20 μM cisplatin, and A2780CP and A2780s cells were treated with 15 μM cisplatin. After exposure to the drugs for 48 h, the cells were dissociated using 0.05% EDTA-free trypsin and washed with cold PBS. Approximately 1 × 10^6^ cells were suspended in 100 µL of Annexin V incubation reagent. After incubation in the dark for 20 min at room temperature, cellular fluorescence was analyzed by the BD FACSCalibur Flow Cytometer (BD Biosciences, USA) within 30 min.

### Tumor cell wound healing assay

After growth to 70%–80% confluence in 6-cm cell culture dishes, the cells were incubated with cisplatin (10 μM for A2780s and 15 μM for OV2008) for 12 h, and then the cell monolayer was scratched across the center of the wells using a 10 μL pipette, washed twice with MEM, and cultured in MEM without FBS for up to 48 h. At the end of each experiment, images were captured using an Olympus IX73 microscope connected to an Olympus DP73 camera (Olympus corporation, Tokyo, Japan) at 0, 8, 16, and 24 h.

### Tumor cell invasion assay

To analyze the invasion capacity, we first coated cell inserts (8.0 μm pore size membrane; Corning, Corning, NY, USA) with 100 μL of Matrigel (at a dilution of 1:3 in DMEM) (BD Bioscience, San Jose, CA, USA). The cells were serum starved for 24 h, and then 2 × 10^4^ cells were seeded onto the cell inserts in the upper chamber in serum-free medium with 10 μM or 20 μM cisplatin. The bottom chamber, which contained 50% DMEM with 10% FBS and L-glutamine with 10 μM or 20 μM cisplatin, was used as a chemoattractant. After 24 h, cells that invaded the lower surface of the filter were fixed with 4% paraformaldehyde, stained in 0.5% crystal violet, and counted using a microscope. Each experiment was performed in triplicate.

### Protein extraction and western blotting (WB)

Total protein lysates were prepared using standard RIPA lysis buffer (Sigma-Aldrich) with proteinase and phosphatase inhibitors (Santa Cruz Biotechnology, Santa Cruz, CA, USA). The total protein content was estimated by the Pierce™ BCA Protein Assay Kit (Thermo Scientific) following the manufacturer’s protocol. Protein lysates (50 μg) were separated by 12% sodium dodecyl sulfate–polyacrylamide gel electrophoresis (SDS-PAGE) and transferred onto polyvinylidene fluoride (PVDF) membranes (Millipore, Billerica, MA, USA). The membranes were blocked in 5% skim milk solution in TBST buffer for 1 h at approximately 24 °C and incubated with polyclonal Connexin 43 (1:500; Proteintech Group, Wuhan, China) and polyclonal GAPDH (1:2000; Proteintech Group, Wuhan, China) primary antibodies at 4 °C overnight. The membranes were incubated with horseradish peroxidase (HRP)‐conjugated secondary antibody (1:5000, GE, HyClone) at 37 °C for 2 h The protein bands were visualized with enhanced chemiluminescence (ECL; Advansta) and detected using a ChemiDocTM MP imaging system (Bio‐Rad). The protein bands were then scanned using Image LabTM Software Version 4.1.

### In vivo tumor xenograft study

Twenty female SCID mice (approximately 20 mg) were purchased from the Experimental Animal Center of Dalian Medical University. The research protocol was approved, and the mice were maintained in accordance with the institutional guidelines of the Committee on the Use and Care on Animals (Dalian Medical University, Dalian, China). Ovarian cancer cell lines stably transfected with p-EGP-miR-206 (A2780s-206, OV2008-206) and vector control (A2780s-con, OV2008-con) plasmids were injected (5 × 10^6^ cells per mouse in 200 µL) subcutaneously into the left axilla or back of 20 female SCID mice. The tumors were allowed to grow to approximately 100 mm^3^ for A2780s and 30 mm^3^ for OV2008, and then the mice were given 4 mg/kg cisplatin through intraperitoneal injection once every 3 days. Tumor volume in SCID mice was measured every two days before and after cisplatin treatment. The animals in the A2780s group were sacrificed at 21 days after tumor implantation, and the animals in the OV2008 group were sacrificed at 28 days after tumor implantation to confirm the presence of tumors and weigh the established tumors.

### Statistical analysis

The Mann–Whitney U test was performed for qRT-PCR statistical analysis. The data are expressed as the arithmetic mean ± SD of the number (n) of experiments. The samples were analyzed with repeated measures analysis of variance, and differences in incidences were analyzed using one-way ANOVA or t test via SPSS software and GraphPad Prism 8. Overall survival was defined as the time from initial cytoreductive surgery to the date of the last follow-up or death. Survival time courses were evaluated using the Kaplan–Meier method, and the groups were compared using the log rank test. Receiver operating characteristic (ROC) curve analysis was performed for selected miRNAs. In addition, the area under the curve (AUC) values and 95% confidence intervals (CIs) were calculated to evaluate the specificity and sensitivity for predicting chemosensitivity to cisplatin. P < 0.05 was considered significant.

## Results

### miR-206 was significantly increased in EOC with IR, and high miR-206 correlated with poor prognosis in EOC patients

**Fig. 1:**
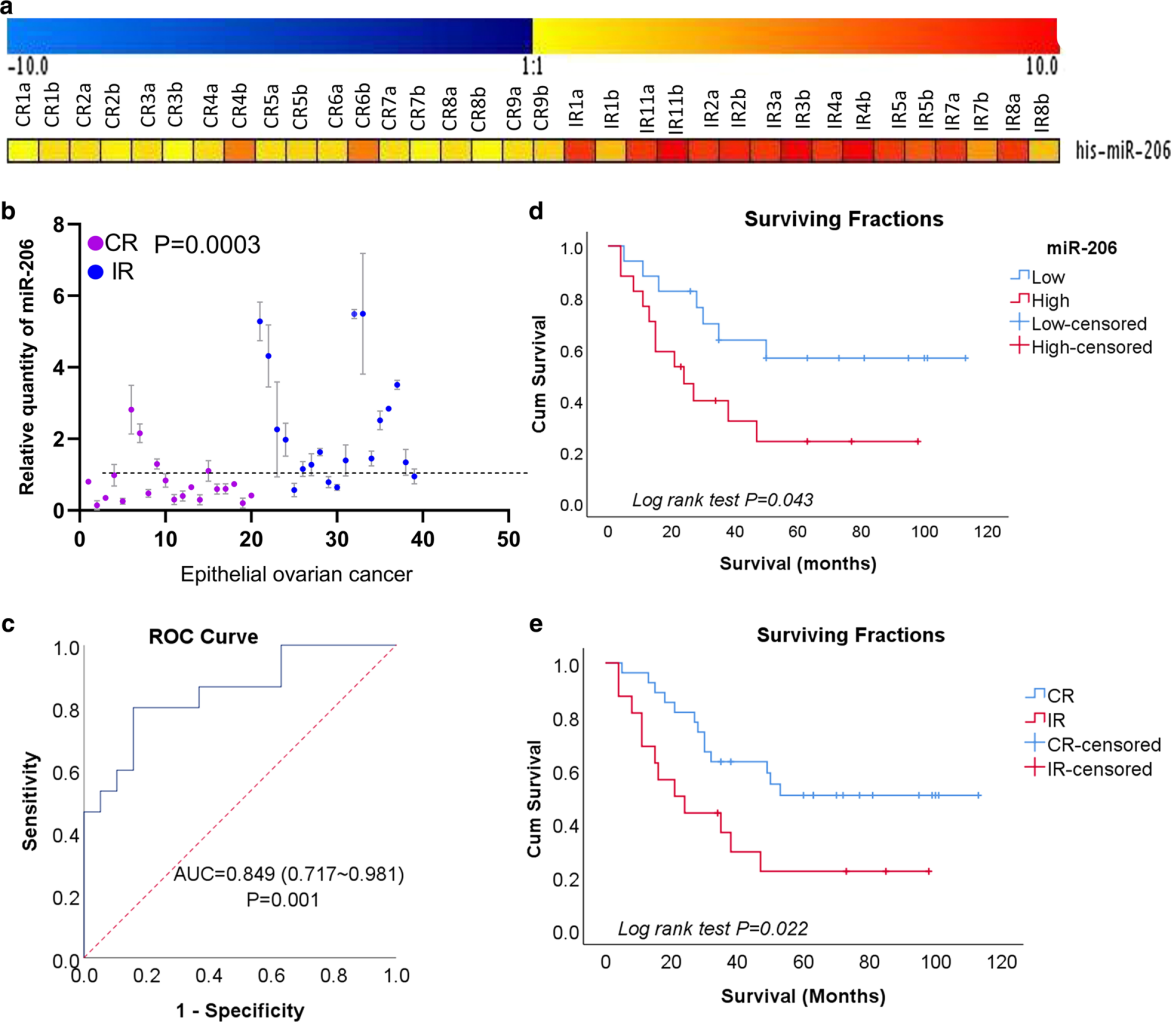
miR-206 levels predict the response to platinum-based chemotherapy and survival in Chinese EOC patients. **a** Hierarchical clustering of miRNA microarray analysis between epithelial ovarian cancer (EOC) samples with complete response (CR) and incomplete response (IR) to platinum-based chemotherapy (n = 17) (P < 0.0001). **b** miR-206 expression in a verified cohort of EOC patients (n = 39) by qRT-PCR. miR-206 was significantly upregulated in the IR group (n = 19) vs. the CR group (n = 20) (P = 0.0033). **c** Receiver operating characteristic (ROC) curve of the cisplatin responders. A Mann–Whitney U test demonstrated that miR-206 expression distinguished IR patients from CR patients (P < 0.001). The area under the curve (AUC) is 0.829 (0.707–0.951). **d** Kaplan–Meier curves showing the overall survival of EOC patients stratified by expression levels of miR-206. The qRT-PCR data of miR-206 was separated into high and low expression groups by the median value. (n = 20 for CR and n = 19 for IR, P < 0.001). **e** Kaplan–Meier curves showing that the response to chemotherapy was related to patient prognosis

To identify the unique miRNA expression pattern associated with resistance to platinum-based chemotherapy in EOC patients, we applied miRNA microarray technology to EOC specimens from CR and IR patients. Of the 768 miRNAs analyzed by microarray, 39 miRNAs showed at least a twofold difference (P < 0.05) between CR and IR patients. Thirty-four miRNAs were downregulated and 5 were upregulated in the IR specimens [2]. Among these differentially expressed miRNAs, miR-206 was one of the most upregulated miRNAs in the IR group compared with the CR group (expression ratio of IR group to CR group = 10.13, P = 1.59E−06, adjusted P = 1.07E−03) (Fig. 1a).

Then, the microarray results were validated by qRT-PCR with another 39 fixed specimens from EOC patients with complete clinicopathological data and survival information. These specimens contained 19 IR EOC cases and 20 CR EOC cases (Table 1). Consistent with the microarray results, miR-206 was significantly upregulated in the IR EOC samples compared with the CR EOC samples (Fig. 1b) (P = 0.0003). To explore the diagnostic potential of miR-206 for chemoresponsiveness, ROC curves were constructed (Fig. 1c). The area under the ROC curve (AUC) for miR-206 was 0.811 (95% CI 0.677–0.93344, P = 0.007), showing relatively high accuracy in chemoresponsiveness prediction. The patient response to platinum-based therapy was accurately predicted in 31 of 39 patients for an overall accuracy of 79.48% (Fig. 1b). A Mann–Whitney U test for statistical significance (P < 0.001) confirmed that this predictor distinguished IR patients from CR patients.

The correlations between miR‑206 expression and other clinicopathological features, including age (> 50 or ≤ 50 years), lymph node metastasis, grade and FIGO stage, were analyzed. miR-206 expression was first separated into high and low expression defined by reference to the median value of the miRNA in the tumor samples. Chi-square tests showed that the expression of miR-206 was not associated with these features except chemosensitivity (Table 2).

**Table 2:** Correlation between the miR-206 expression and clinicopathologic parameters of patients with epithelium ovarian cancer

Clinicopathologic parameters	N	miR-206 expression		P
		Low	High	
Age in years				0.253
< 50	18	10	8	
≥ 50	21	10	11	
Subtypes				
LGSC	4	2	2	0.229
HGSC	35	18	17	
FIGO stage				0.183
I	2	0	2	
II	3	2	1	
III	32	18	14	
IV	2	0	2	
LN metastasis				0.268
Absent	13	7	6	
Present	26	14	12	
Chemotherapy				
CR	20	16	4	*0.001*
IR	19	4	15	

N, number; FIGO stage, International Federation of Gynecology and Obstetrics stage; P, P-value; LGSC, low grade serous carcinoma; HGSC, high grade serous carcinoma; CR, complete response; IR, incomplete response

The Ps set in italic considered statistically significant at < 0.05

To further investigate the role of miR-206 in clinical progression, the patients were followed up, and the clinicopathological data were used in the survival analysis. Kaplan–Meier survival analysis revealed that high miR-206 expression was associated with shorter overall survival (Fig. 1d, Table 3). In addition to miR-206, FIGO staging and sensitivity to chemotherapy (Fig. 1e) were also shown to be associated with survival (Table 3). This result supports the hypothesis that miR-206 may play an important biological role in mediating survival in cancers by modulating the response to chemotherapy. miR-206 may serve as an effective predictor of platinum chemosensitivity and survival in patients with EOC.

**Table 3:** Univariate analysis of overall survival in patients with epithelial ovarian cancer

	Number	Estimate	Std. error	95% Confidence interval		P (Log Rank, Mantel-Cox)
				Lower bound	Upper bound	
Age						0.572
≤ 50	17	64.900	10.051	45.200	84.600	
> 50	22	52.803	8.234	36.665	68.941	
Chemotherapy sensitivity						*0.022*
CR	20	71.410	8.417	54.913	87.907	
IR	19	37.313	8.808	20.048	54.577	
miR-206 expression						*0.043*
Low	20	74.923	11.042	53.280	96.566	
High	19	38.825	8.969	21.246	56.404	
Subtype						0.245
LGSC	4	74.666	10.091	59.289	93.044	
HGSC	35	62.623	8.678	45.613	79.632	
FIGO stage						*0.016*
I	2					
II	3	75.500	3.889	67.877	83.123	
III	32	56.921	7.655	41.917	71.925	
IV	2	12.500	8.500	0.000	29.160	

FIGO stage, International Federation of Gynecology and Obstetrics stage; HGSC, high grade serous carcinoma; LGSC, low grade serous carcinoma; CR, complete response; IR, incomplete response; P, P-value

The Ps set in italics considered statistically significant at < 0.05

**Fig. 2:**
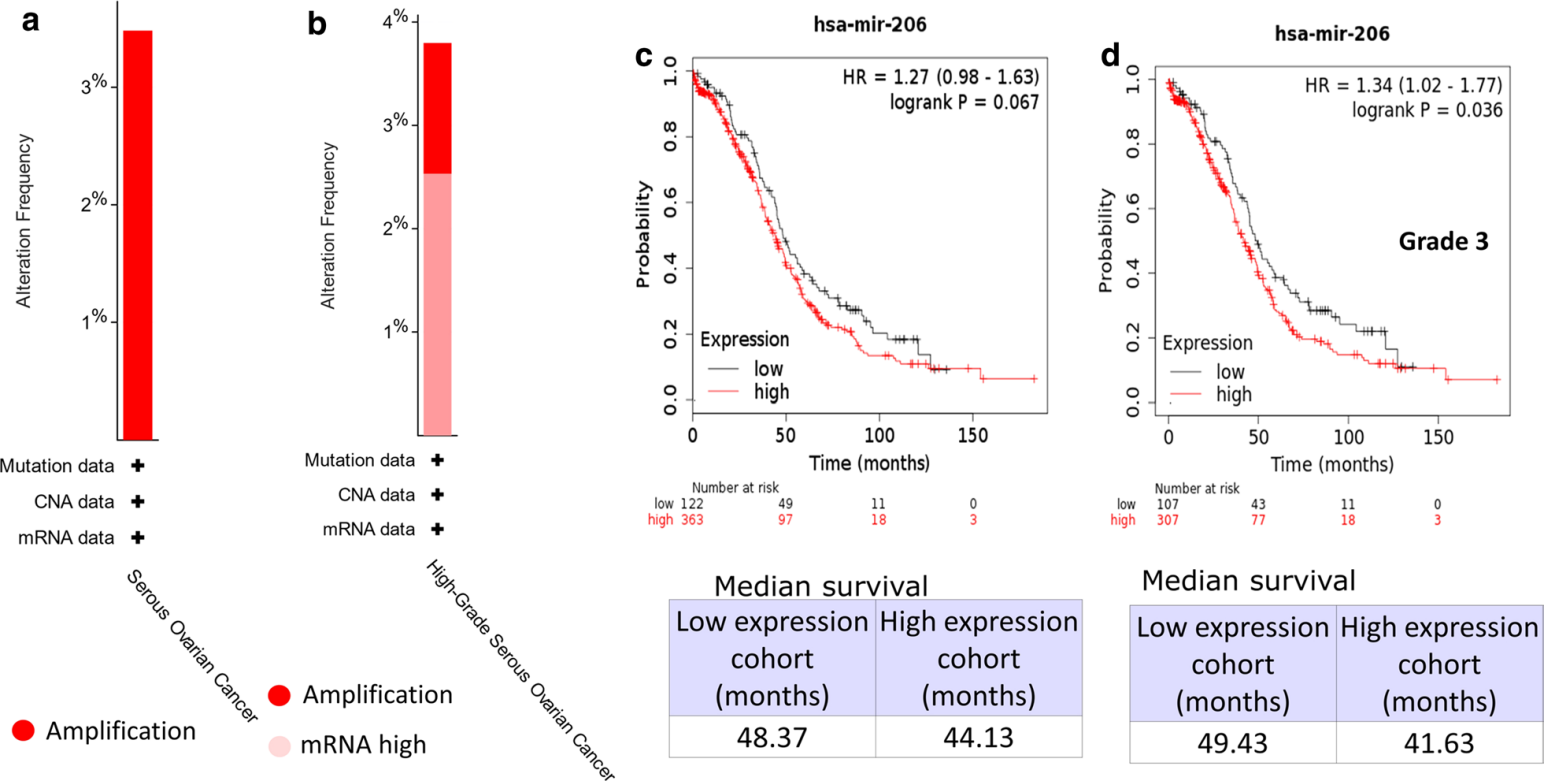
Genetic alterations and survival analysis of miR-206 in ovarian cancer. **a**, **b** Graphs indicate the frequency of miR-206 somatic mutations in ovarian cancers extracted from cancer studies in TCGA (TCGA, PanCancer Atlas (**a**) and TCGA, Nature 2011 (**b**)). **c**, **d** Kaplan–Meier diagrams showing the overall survival of patients with ovarian cancer (**c**) and grade 3 ovarian cancer (**d**) depending on the expression of miR-206 in kmplot.com

The somatic mutations and prognostic significance of miR-206 were further investigated in some current widely used databases. The genetic alterations identified by The Cancer Genome Atlas (TCGA) as affecting miR-206 in OSC are shown in Fig. 2a and b, which indicate a 3% amplification of the gene in a 201 OSC patient cohort (TCGA, PanCancer Atlas) and 1.5% amplification of the gene in a 316 OSC patient cohort (TCGA, Nature 2011). No other genetic alterations, such as deletions or mutations, were detected. Survival analysis by KmPlot [13] showed that in ovarian cancer, patients with low miR-206 expression appeared to have a longer median survival time (49.43) than those with high miR-206 expression (44.53); however, the difference was not significant (P = 0.068) (Fig. 2c). We then analyzed the prognostic significance of miR-206 in EOC patients with different races, grades, and mutation burdens (Additional file 1: Fig. S1) and found that miR-206 expression was significantly associated with poor prognosis in high-grade (grade 3) EOCs (P = 0.036) (Fig. 2d).

### Overexpression of miR-206 increased the chemotherapy resistance of EOC to cisplatin

**Fig. 3:**
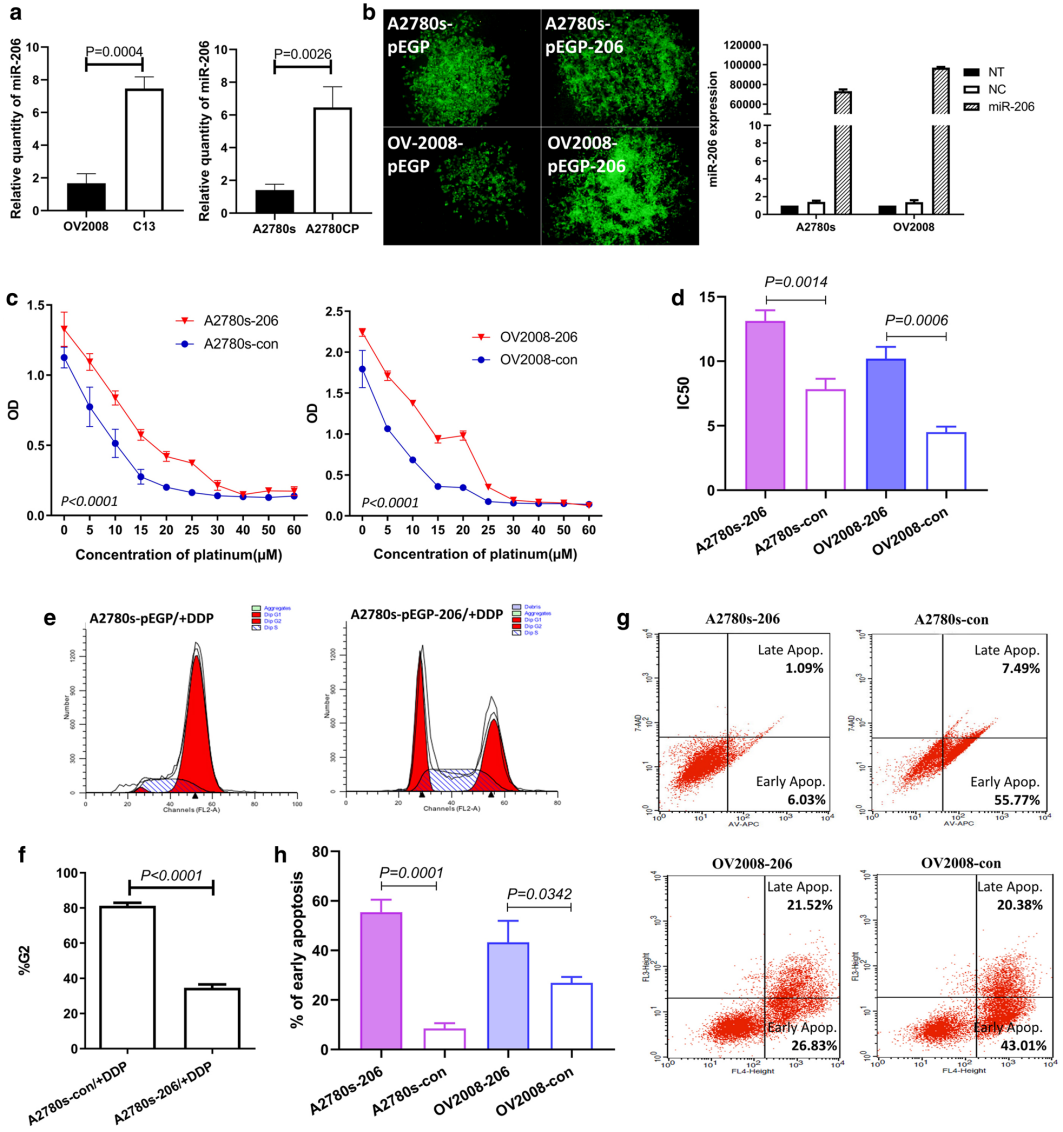
Overexpression of miR-206 increased the resistance of EOC to cisplatin. A2780s and OV2008 cells were stably transfected with pre-miR-206 or vector control and then subjected to different assays. **a** qRT-PCR showed miR-206 expression in parental cisplatin-sensitive EOC cell lines A2780s and OV2008 and their cisplatin-resistant variants A2780CP and C13. **b** qRT-PCR showed miR-206 expression in various EOC cell lines. **c** The MTS results show the dose–response curve of cisplatin. **d** The IC50 values are shown. **e** Flow cytometry results showed the comparison of the cell populations in the G0/G1, S and G2/M phases between the A2780s and A2780s-206 groups. **f** Bar graphs showing the percentage of cells in the G2 phase detected by flow cytometry. **g** Annexin V flow cytometry assessment of the apoptotic cell population after 24 h of cisplatin treatment. **h** The percentage of cells in early apoptosis

To further verify the relationship between miR-206 expression and responsiveness to platinum-based chemotherapy in ovarian cancer, two pairs of parental EOC cell lines, A2780s and OV2008, known as cisplatin-sensitive, and their cisplatin-resistant variants A2780CP and C13 were used for in vitro experiments. The qRT-PCR results showed significantly higher miR-206 expression levels in C13 and A2780CP cells than in their cisplatin-sensitive counterparts. These results are consistent with those of the clinical EOC patient specimens (Fig. 3a).

To further investigate whether miR-206 is functionally involved in cellular sensitivity to cisplatin, the expression vector pEGP-miR (5.0 kb) carrying pre-miR-206 was transfected into two cisplatin-sensitive cell lines, OV2008 and A2780s, which have low miR-206 expression. After two weeks of antibiotic selection, miR-206 was confirmed to be overexpressed in the stably transfected cell lines (OV2008-206 and A2780s-206). miR-206 expression was hundreds-fold higher in OV2008-206 and A2780s-206 cells than in empty vector-transfected controls (A2780s-con and OV2008-con) (Fig. 3b).

We then investigated the effects of miR-206 overexpression on sensitivity to cisplatin chemotherapy in sensitive cell lines. The cells were treated with different concentrations of cisplatin. The MTS results showed that A2780s-206 and OV2008-206 cells were more resistant to cisplatin than the controls (A2780s-con, OV2008-con) (Fig. 3c). The drug concentrations that inhibited cell growth by 50% (IC50 values) in the A2780s-206 cells and OV2008-206 cells were ~ 1.5-fold and 2.5-fold higher, respectively, than those in their controls (P < 0.01) (Fig. 3d). These data indicated that forced miR-206 expression promoted cisplatin resistance in EOC cell lines.

Cisplatin (DDP) and other platinum-based cancer drugs bind to double-stranded DNA and form DNA adducts, interfering with DNA replication and RNA transcription and ultimately triggering apoptosis. We then examined the effect of miR-206 overexpression on cisplatin-induced cell cycle changes and apoptosis. After the cells were treated with cisplatin for 24 h, flow cytometric data indicated that miR-206 overexpression promoted cell cycle progression and decreased apoptosis during cisplatin treatment. Compared with that in the negative control, the cell cycle G2 phase ratio was decreased, and the G1 and S phase ratio was increased in A2780s-206 cells (Fig. 3e, f). The proportion of A2780s-206 and OV2008-206 cells in early apoptosis was significantly lower than that of the negative controls (Fig. 3g, h). These data indicated that miR-206 overexpression significantly decreased the cytotoxicity caused by cisplatin in A2780s and OV2008 cells.

### miR-206 abrogated the suppressive effect of cisplatin on migration and invasion in cisplatin-sensitive ovarian carcinoma cells

The progression of a malignant tumor is determined by the metastatic and invasive properties of tumor cells, which allow malignant cells to invade the extracellular matrix and metastasize to distant sites. We investigated the effect of miR-206 on the migration and invasion ability of EOC cells, especially during primary chemotherapy.

**Fig. 4:**
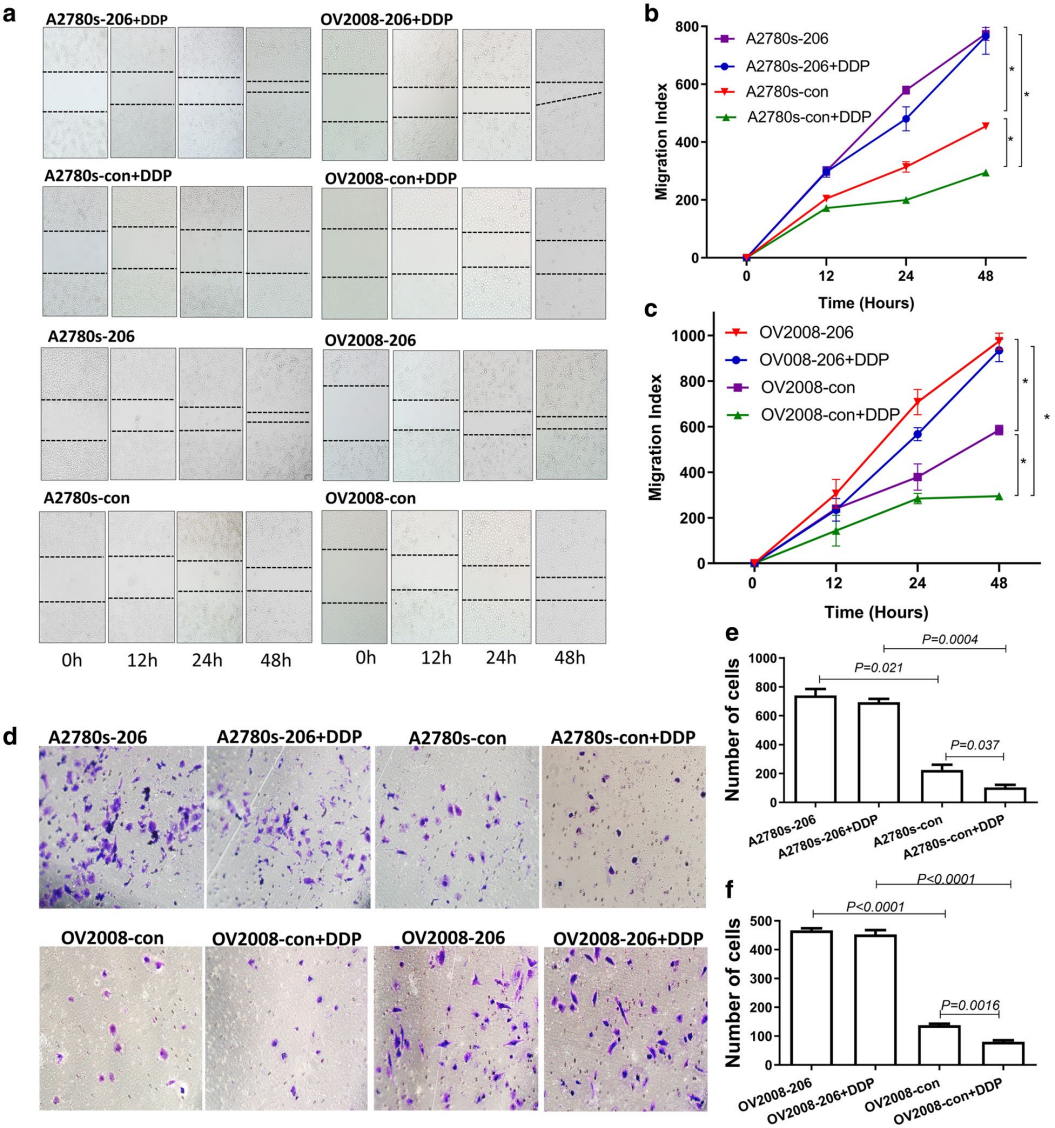
Overexpressed miR-206 promotes migration and invasion by effectively abolishing the function of cisplatin. A2780s and OV2008 cells were stably transfected with p-EGP-miR-206 plasmids or vector control plasmids and then subjected to different assays. **a** Representative microphotographs of the wound healing assay. **b**, **c** Quantification of the wound healing assay data. **d** Representative microphotographs of the tumor cell invasion assay. **e**, **f** Quantification of the cell invasion assay of EOC cell lines

The wound healing assay showed that the speed of migration of both OV2008-206 and A2780s-206 cells was significantly faster than that of the corresponding control cells A2780s-con (P < 0.001) and OV2008-con (P < 0.001), which suggested that miR-206 promotes migration in these EOC cells (Fig. 4a–c). When the cells were treated with cisplatin, consistent with their cisplatin-sensitive nature, the migration of the two cell lines A2780-con and OV2008-con was significantly decreased (P = 0.034, P = 0.0025). However, for the cells with stably enforced miR-206 expression (OV2008-206 + DDP and A2780s-206 + DDP), there was no significant change in the migration index compared with the corresponding untreated cell lines OV2008-206 and A2780s-206 (P > 0.05) (Fig. 4a–c).

Invasion ability was tested using quantitative Boyden chamber analysis. Similar to the results of the wound healing assay, miR-206 significantly promoted the invasion of EOC cells, as shown by the finding that the number of transferred cells in the OV2008-206 and A2780s-206 cell lines was greater than that in the OV2008-con (P < 0.0001) and A2780s-con (P = 0.021) cells (Fig. 4d–f). When the cells were treated with cisplatin, the invasion ability was clearly inhibited in the cisplatin-sensitive cell lines OV2008-con and A2780s-con (P < 0.05), while for cells with high miR-206 expression (A2780s-206 and OV2008-206), cisplatin treatment did not affect the number of cells transferred to the other side of the chamber (P > 0.05) (Fig. 4d–f). These data indicated that miR-206 abrogated cisplatin-reduced migration and invasion and reduced the cytocidal effect of cisplatin.

### miR-206 promoted cisplatin chemoresistance by directly targeting Connexin 43 (Cx43)

**Fig. 5:**
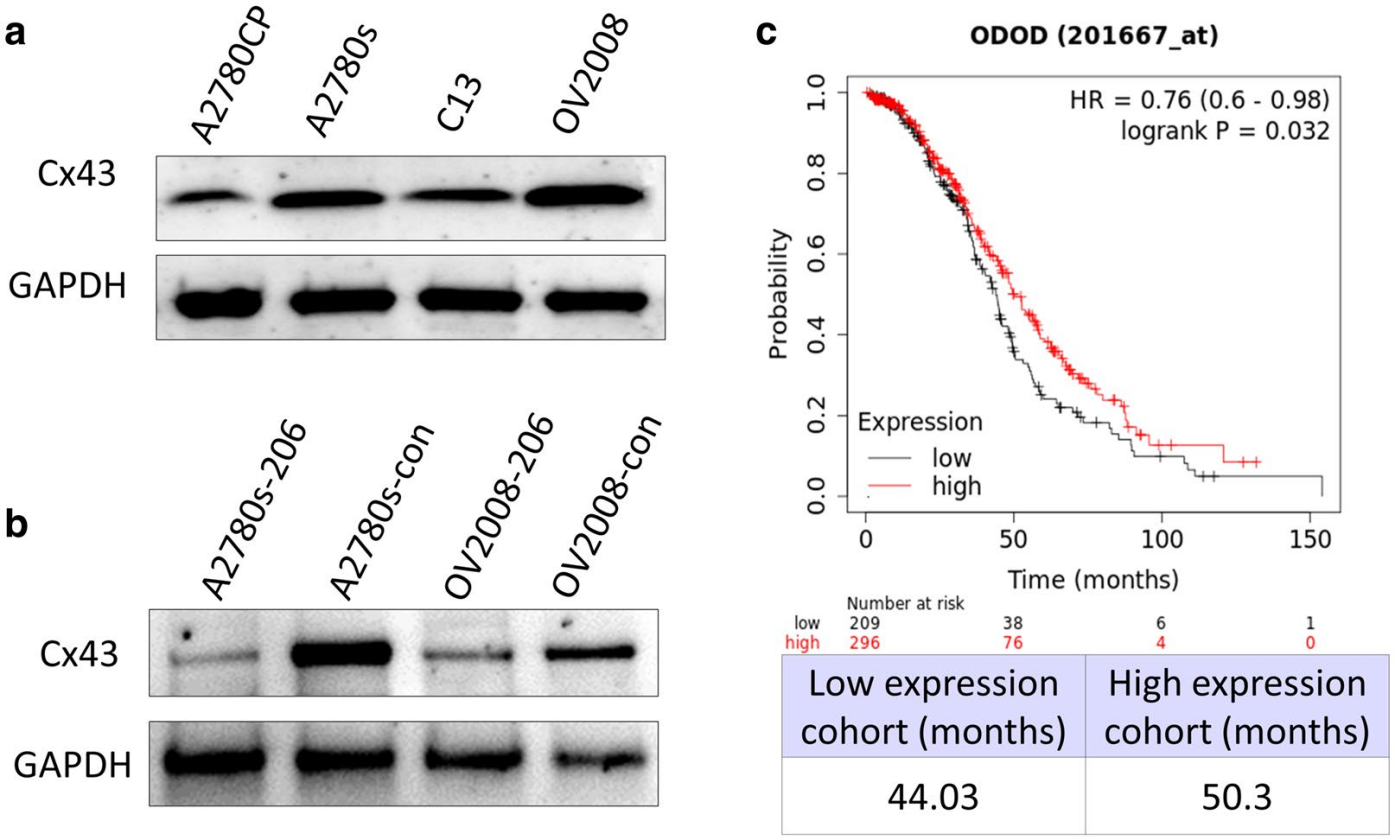
miR-206 targets Cx43. **a** Western-blot detection of the expression of Cx43 protein in ovarian cancer cell lines. **b** Western-blot detection of the expression of Cx43 protein after up-regulation of miR-206. **c** Kaplan–Meier diagrams showing the overall survival of EOC patients who received platinum chemotherapy depending on the expression of Cx43 mRNA in kmplot.com

Cx43 has been found to be a direct target of miR-206 in many cells, including smooth muscle cells [14], myocardial cells [15], type II alveolar epithelial cells [15] and breast cancer cells [16]. Cx43 is a gap junction protein that promotes cisplatin cytotoxicity [17]. To investigate whether miR-206 promotes cisplatin chemoresistance by targeting Cx43 in EOC, we examined the effect of miR-206 on the expression of Cx43 in EOC cell lines. Western blot analysis showed that miR-206 overexpression significantly reduced the expression of Cx43 in the A2780s and OV2008 cell lines (Fig. 5a). Then, we examined the relationship between Cx43 expression and platinum chemosensitivity in ovarian cancer cell lines. The expression of Cx43 in two platinum-sensitive EOC cell lines, A2780s and OV2008, was significantly higher than that in their cisplatin-resistant variants, A2780CP and C13 (Fig. 5b), which is the opposite of the expression of miR-206 in these cells. Survival analysis of data from TCGA by KmPlot showed that high Cx43 miRNA expression was associated with better overall survival in patients who received platinum chemotherapy (P = 0.032) (Fig. 5c).

### miR-206 promoted cisplatin chemoresistance in EOC in vivo

**Fig. 6:**
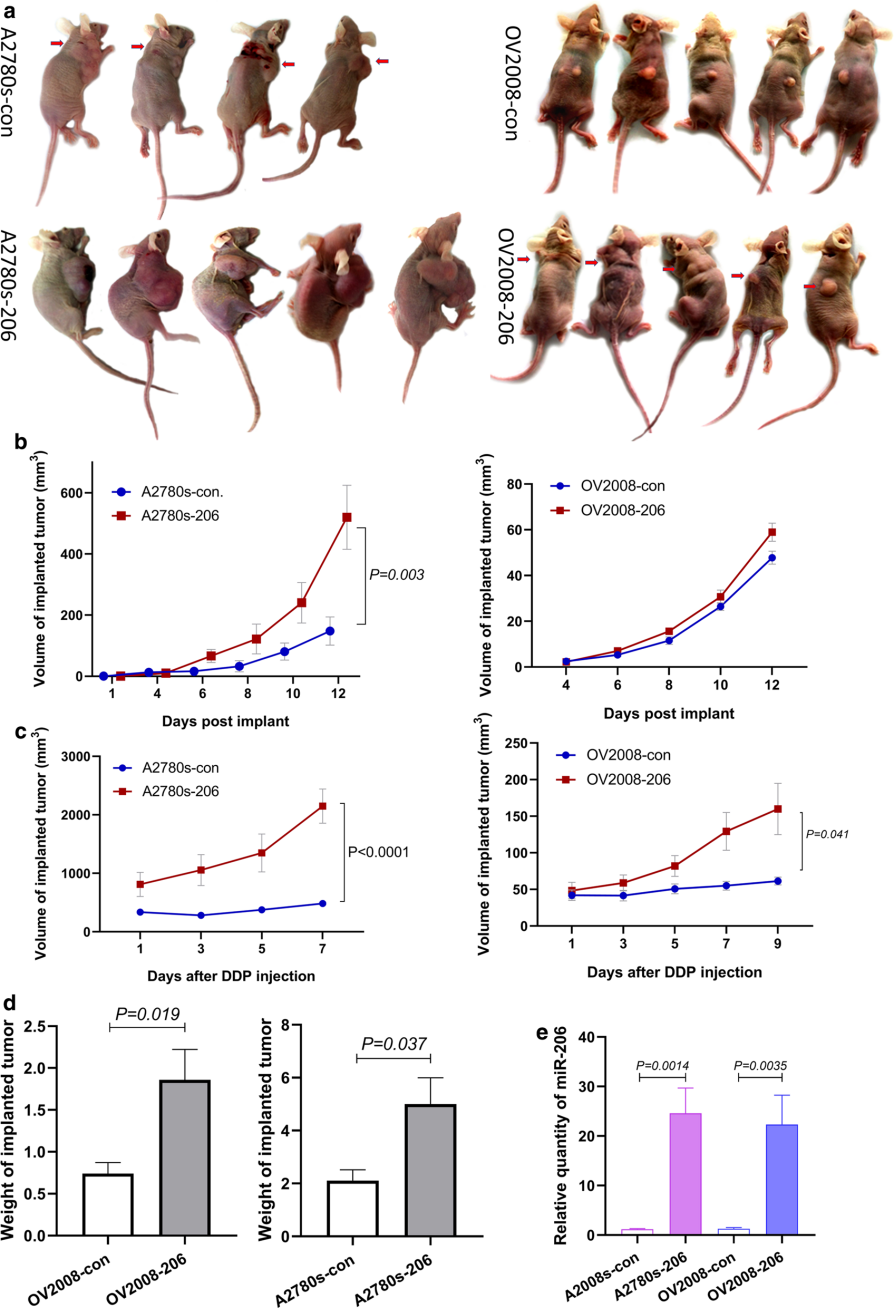
miR-206 enhances EOC chemoresistance to cisplatin in vivo. Xenograft tumor growth derived from subcutaneously injected EOC cells stably transfected with p-EGP-miR-206 plasmids (A2780s-206 and OV2008-206) or vector control plasmids (A2780s-con and OV2008-con) in 20 female SCID mice. Tumors were allowed to grow to approximately 100 mm3 for A2780s and 50 mm3 for OV2008, and then the mice were given 4 mg/kg cisplatin through intraperitoneal injection once every 3 days. **a** Tumors developed in the four groups of mice before the dissection of tumors. **b** Tumor volume in SCID mice was measured every two days before cisplatin treatment (some tumors are indicated by arrows). **c** Tumor volume in SCID mice was measured every two days after cisplatin treatment. **d** Tumor weight of SCID mice at the termination of the experiments. **e** qRT-PCR showed miR-206 expression in implanted tumors derived from EOCs stably transfected with miR-206 and empty vector

To explore the role of miR-206 in cisplatin resistance in vivo, we used a subcutaneous injection model in immune-deficient nude mice. A2780s-206 and OV2008-206 cells and their corresponding controls were injected subcutaneously into nude mice, and the tumors were allowed to grow for approximately 2 weeks. When the implanted tumors reached a certain size, the mice were treated with cisplatin at 4 mg/kg via intraperitoneal injection 2 times per week for 1 to 2 weeks (Fig. 6a). The tumor growth rate was recorded.

Tumors derived from A2780s-206 cells developed much earlier and grew faster than those in the control group (Fig. 6a, b). After cisplatin treatment, the A2780s-con tumors ceased growing, some tumors began to shrink, and the tumors were confined to the site of implantation, while the A2780s-206 tumors continued growing and diffused to different parts of the body (Fig. 6a, c). Cachexia in mice from advanced carcinoma was clearly observed in A2780s-206 nude mice after 2–3 treatments, and the cachexia symptoms developed even earlier and to a greater degree in A2780s-206 mice injected with cisplatin.

The same experiment was performed using OV2008 cells. Before chemotherapy, although the size of OV2008-206 tumors grew slightly larger than that of OV2008-con tumors, there was no significant difference in tumor size between the two groups (Fig. 6a, b). After cisplatin treatment, consistent with the results of A2780s, cisplatin profoundly diminished the volume of the implanted tumors derived from OV2008-con cells, while it exerted no observable effect on the implanted tumors derived from OV2008-206 cells (Fig. 6a, c). These results demonstrated that upregulation of miR-206 significantly promoted the chemoresistance of EOC to cisplatin treatment.

After chemotherapy, samples were collected, and the tumor sizes and weights were measured. The results showed that the volume of tumors derived from miR-206-upregulated cell lines (A2780s-206, OV2008-206) was significantly larger (P < 0.001) than that of tumors derived from the controls (A2780s-con, OV2008-con) (Fig. 6d). miR-206 expression in the tumors was verified by qRT-PCR. The results showed that miR-206 expression in tumor tissue derived from A2780s-206 and OV2008-206 cell lines was 20.99 ± 2.41 and 17.64 ± 4.67 times higher than that in tumors derived from the vector control cells (Fig. 6e). These results strongly suggested that miR-206 enhanced resistance to cisplatin chemotherapy in EOC.

## Discussion

The platinum-taxane doublet represents the gold standard treatment in EOC [18]. However, not all EOCs respond well to platinum-based regimens. The initial response rates are 60–80%; eventually, however, the majority of patients become platinum-resistant and experience subsequent relapse. More than 20% of ovarian cancer patients with platinum-resistant tumors do not benefit from first-line platinum-based chemotherapy. The identification of reliable biomarkers enabling clinicians to distinguish IR from CR subjects before chemotherapy is one of the main goals of translational medicine.

In recent years, enormous efforts have been made to identify effective biomarkers that can predict sensitivity to platinum-based chemotherapy in EOC. A subset of miRNAs has been found [2, 19–25], and only a few miRNAs overlap (Additional file 2: Table S1). Further validation is still needed. By microarray, we identified a spectrum of miRNAs that significantly differentiated IR samples from CR samples [2]. Among these miRNAs, our group validated several of the most significant miRNAs, including miR-770-5P [2], miR-136 [10], miR-224-5p [18] and miR-206, by functional studies and qRT-PCR in additional patient cohorts. We believe that a single miRNA or a combination of these miRNAs may be used as a biomarker to predict chemosensitivity to platinum drugs.

Among the dysregulated miRNAs, miR-206 was one of the most significantly upregulated miRNAs in EOC samples from IR patients compared with those from CR patients. This result was further confirmed in a validation cohort including 39 cases of EOC and two pairs of corresponding EOC cell lines. Retrospective assessments of miR-206 as a predictor of primary chemosensitivity showed that high miR-206 expression predicted chemoresistance with an accuracy of up to 79.48%. These findings suggested that miR-206 expression levels can be used as a possible biomarker for the therapeutic response to platinum-based chemotherapy in EOC and may be used as a biomarker to assist in selecting the appropriate chemotherapy regimen before treatment begins, helping to avoid unnecessary toxicities and enhance quality of life. Moreover, our results also showed that high miR-206 expression correlated with unfavorable survival outcomes in EOC patients. Using data from KmPlot, we found that high miR-206 expression was related to poor overall survival in high-grade EOCs. These data suggested that miR-206 may also be a valuable prognostic predictor, especially for high-grade EOCs.

To further investigate the functional effect of miR-206 on platinum-based chemotherapy, we stably enforced miR-206 expression in platinum-sensitive EOC cell lines. In vitro experiments showed that overexpression of miR-206 in cisplatin-sensitive EOC cell lines significantly increased cell viability, migration and invasion in the presence of cisplatin and decreased cisplatin-induced apoptosis. In vivo animal experiments showed that miR-206 promoted the growth of tumors derived from A2780s-206 cells but not OV2008-206 cells. The miR-206-upregulated tumors derived from both cell lines were more resistant to cisplatin treatment. The in vivo study suggested that miR-206 may not promote proliferation in EOC cell lines, but it definitely enhanced the chemoresistance of the cells to cisplatin. The results of both in vitro and in vivo experiments indicated that miR-206 plays an important role in enhancing chemoresistance to cisplatin.

miR-206 belongs to the miR-206/miR-133b cluster located in chromosomal region 6p12.2. This cluster is considered myomiRs [26]. miR-206 acts to promote myogenic differentiation [27] and plays a positive role in the regeneration of muscles during injury. The role of miR-206 in cancers is complicated and controversial. Decreased expression of miR-206 was found in rhabdomyosarcoma [26], lung cancer [28], ER + breast cancer [29, 30], renal cell carcinoma [31], ER alpha + endometrioid adenocarcinoma [32], hepatocellular carcinoma [33, 34] and glioma [35]. Upregulation of miR-206 inhibits the migration, invasion and proliferation of breast cancer [30], renal cell carcinoma [31], glioma [35], and head and neck squamous cell carcinoma [31], suggesting a tumor suppressor role of miR-206. Increased expression of miR-206 was found in breast cancer [30, 36], esophageal carcinoma [37] and some soft tissue sarcomas [38], and high miR-206 expression was related to the poor prognosis of breast cancer [36] and esophageal carcinoma patients [37]. Upregulation of miR-206 in breast cancer cells promoted the migration, invasion and proliferation of breast cancer cells [30]. These data indicated that miR-206 can also serve as an oncomiR. It appears that the role of miR-206 in cancers is tissue specific.

The mechanism of miR-206 in platinum resistance remains unclear. Commonly, miRNAs exert effects through their target protein molecules. Many proteins have been reported to be directly targeted by miR-206, including estrogen receptor 1 (ESR1) [6], MET [39], NOTCH 3 [6, 39], HDAC4 [27], Gadd45β [40], and Connexin 43 (Cx43) [14, 15, 41]. Among these targets, Cx43 is a well-defined target molecule of miR-206 in many types of cells, including smooth muscle cells [14], myocardial cells [15], type II alveolar epithelial cells [15] and breast cancer cells [16]. Cx43 is ubiquitous in cells and is reduced in a variety of tumor cells [16]. It influences the response of tumor cells to treatments by facilitating the passage of antitumor drugs or death signals between neighboring tumor cells [17, 42]. Cx43 increased the susceptibility to cisplatin-induced cell death in several tumor types, including melanoma [42], breast cancer [42], adenocarcinoma of the lung [43] and ovarian cancer [44]. In ovarian cancer, Sanjeevani showed that downregulation of Cx43 in A2780s cells decreased the cytotoxicity caused by high-dose cisplatin treatment, and Cx43 enhanced cisplatin cytotoxicity by propagating the “toxic” signals among coupled cancer cells [17]. In this study, we found that upregulation of miR-206 significantly downregulated the expression of Cx43 in EOC cell lines, which confirmed the targeting relationship between Cx43 and miR-206 in EOC. We also found that Cx43 expression was significantly increased in parental cisplatin-sensitive A2780s and OV2008 cells but reduced in the cisplatin-resistant A2780CP and C13 cells. Survival analysis of data from TCGA by KmPlot showed that high Cx43 miRNA expression was associated with better overall survival in patients who received platinum chemotherapy. These data suggested that miR-206 promotes platinum resistance at least partly by downregulating Cx43, which in turn reduces the propagating toxic signals among EOC cells and increases cell survival.

There may be some possible limitations in this study. Korch et al. reported that OV2008 used in some labs was a mislabeled version of the HPV-positive ME-180 cell line [45]. We have done experiments using OV2008 cell line since before 2004 [2, 46–48], but we didn’t retest the identity of the cell line in our lab stocks to make sure it is the ‘‘true’’ HPV-negative 2008 ovarian cancer cell line defined in the report from Korch et al. [45].

## Conclusion

In the present study, we found that high expression of miR-206 predicted platinum resistance and poor prognosis in patients with EOC. miR-206 functionally enhanced platinum resistance in EOC cells. We believe that the miRNA-206 expression profile, either alone or in conjunction with those of other miRNAs, can be used as a novel biomarker to direct adjuvant chemotherapy in patients with EOC prior to treatment. Targeting miR-206 may be a possible way to reverse the cisplatin resistance of EOC.

## Supplementary information

**Additional file 1: Fig S1.** Kaplan–Meier plots showing the overall survival of patients with ovarian cancer according to the expression of miR-206. (a) Overall survival curves are plotted for all ovarian cancer patients of different races. (b) Overall survival curves are plotted for patients with different ovarian cancer mutation burdens.

**Additional file 2: Table S1.** MicroRNAs profile that differently expressed in CR and IR.

## Data Availability

The datasets used and/or analyzed during the current study are available from the corresponding author on reasonable request.
