# Supplementary material for: miR-206 as a prognostic and sensitivity biomarker for platinum chemotherapy in epithelial ovarian cancer
Source: Cancer Cell Int. 2020 Nov 3;20:534. doi: 10.1186/s12935-020-01623-y (PMC7641844; doi:10.1186/s12935-020-01623-y)
Supplement: Supplementary file 1 — Additional file 1: Fig S1. Kaplan–Meier plots showing the overall survival of patients with ovarian cancer according to the expression of miR-206. (a) Overall survival curves are plotted for all ovarian cancer patients of different races. (b) Overall survival curves are plotted for patients with different ovarian cancer mutation burdens. [file 12935_2020_1623_MOESM1_ESM.docx]

**
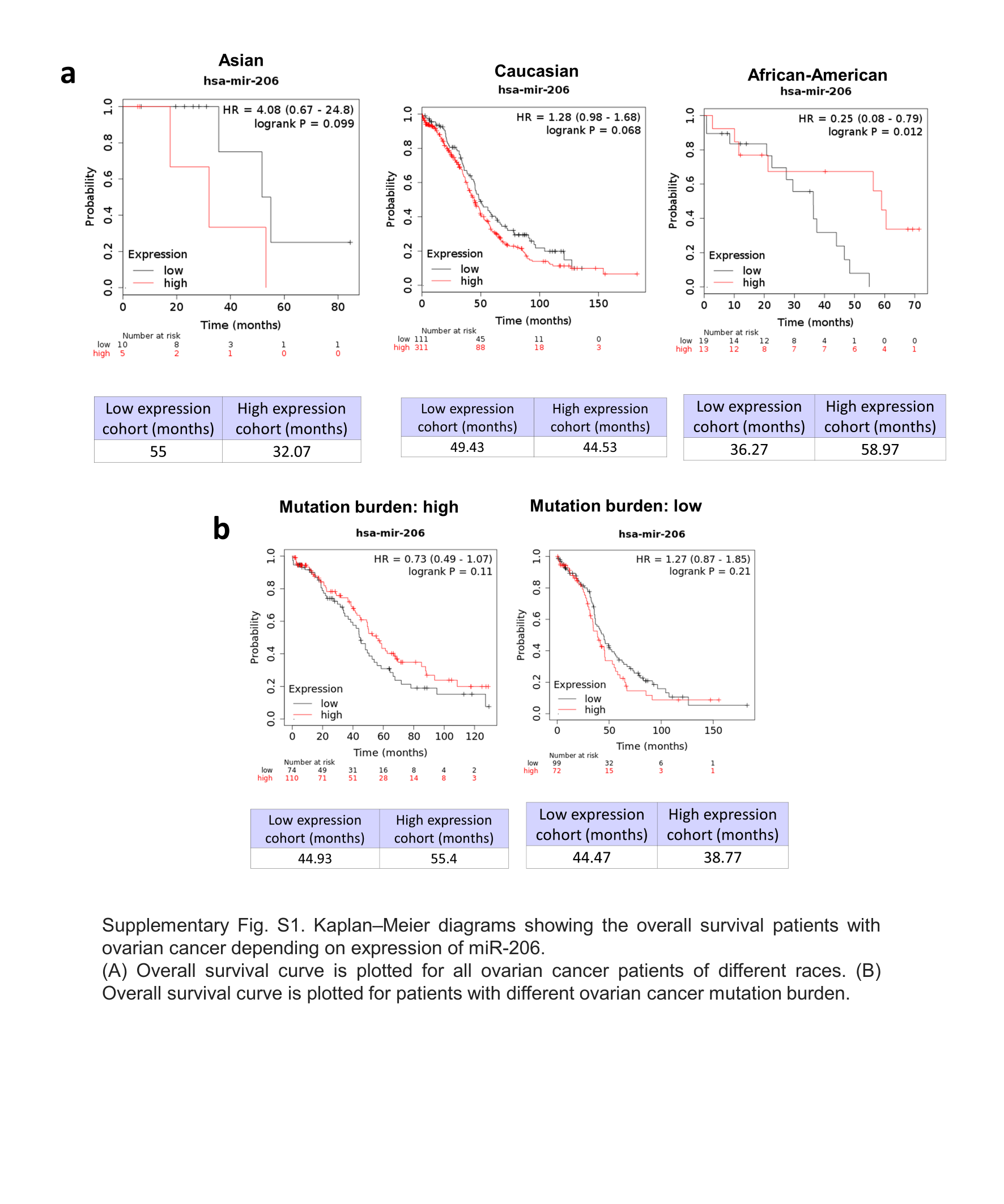
**

**Fig. S1.** Kaplan–Meier plots showing the overall survival of patients with ovarian cancer according to the expression of miR-206.

(a) Overall survival curves are plotted for all ovarian cancer patients of different races. (b) Overall survival curves are plotted for patients with different ovarian cancer mutation burdens.
