# Supplementary material for: miR-206 as a prognostic and sensitivity biomarker for platinum chemotherapy in epithelial ovarian cancer
Source: Cancer Cell Int. 2020 Nov 3;20:534. doi: 10.1186/s12935-020-01623-y (PMC7641844; doi:10.1186/s12935-020-01623-y)
Supplement: Supplementary file 2 — Additional file 2: Table S1. MicroRNAs profile that differently expressed in CR and IR. [file 12935_2020_1623_MOESM2_ESM.docx]

**Table S1. MicroRNAs profile that differently expressed in CR and IR**

| **Reference** | **Sample types** | **Cell type** | **Up-regulated in IR** | **Up-regulated in CR** |
| --- | --- | --- | --- | --- |
| [19] | ovarian cancer specimens CR VS IR | Frozen  41 serous EOC and 31 other types | **miR-29a/ *452*/*126*/ *196a*/ *214/***223/ 216/ 198/ 22/ 370/ 520e/ 519e/ 1b-1/ 152/ 521/ 123/ 365/ 320/ 129/ 146/ 140/ 148/ 320/ 181c/ | **509/ 513/ let-7i**/ 321/ 514/ 507/ 203/ 453/ 491/ 106b/ |
| [2] | ovarian cancer specimens CR VS IR | paraffin-embedded;  serous EOC, 8 IR versus 6 CR | miR-9/***9****/206 | **509-3p/ 513a-3p/ let-7e/ 335/ 218-2/ 493/*130a/ 141* / *452/* *214/*** let-7b/ 133a/ 136/143/ 183/ 200b/ 219-1-3p/ 335*/ 339-5p/ 34b/ 34c-5p/ 376-a/ 376c/ 379/ 380/ 409-3p/ 382/ 411/ 433/ 455-5p/ 485-3p/ 508-3p**/** 539/ 542-3p/ 589/ 770-5p/ 597/ 880-3p/ 80-5P/ |
| [20] | ovarian cancer specimens CR VS IR | paraffin-embedded  35 serous EOC and 21 endometrioid EOC | **miR-27a**/ **23a**/ ***30c***/ ***199a-3p*** /let-7g/ | ***miR-378***/ 625 |
| [21] | SKOV3/CIS VS SKOV3 | fresh cells line | **miR-29a/ 7d / 98/ 27a/23a /*17*** /***let‑7e*** /***let‑7i***/***378* / *106a***/***130a*/ *222* /*19a /20b***  /4284/133b/148b /181d /29b /let‑7c /3175 /let‑7g/ 30b /92a /4289 /744 /320d /3653/ 320a /99a /221 /15a /365 /98 /let‑7f /374 /423‑5p/ 26b /20a /25 /30e /320e /22 /16 /374b /423‑3p /331‑3p /224 /106b /3607-3p /15b/1290 /3651 / | 513a‑5p/***let-7i****/ ***9****/ 630/ 21*/ 205*/ 498/ 31/ 210/ 584/ 1265/ 4308/ 1264/ 4324/ 375/ 3074‑3p/ 7a-2*/ 208a/ 2116/ 1321/ 668/ 1913/ 24‑1*/ 2115*/ 3686/ 1973/ 1284/ 3182/ 193b*/ 3667‑5p/ 181a/ 3926/ 664/ 615‑3p |
| [22] | A2780/cis VS A2780 | fresh cell line | **miR-29a/ 7d / 98/ *10b/*** miR-21/ Let-7a/10a/ 422a/ 27b/ 93/ 190 |  |
| [23] | A2780/DDP VS A2780 | fresh cell line | **miR-141** | ***miR-199a-5p*** /**645**/**335** /**199a-3p/18b /199b-3p**/215 /363 |
| [24] | A2780/cis VS A2780 | fresh cell line | **miR-141**/ 200c/412 | **miR-199a-5p/645**/ **335**/ **199a-3p/18b/ 20b/ 196a/ 106a/493/ 10b/ 19a/** **130a/ 222/ *17***/215/ 338-5p/ 135b/ 186/ 942/ 421/ 19b/ 518e/ 631/ |
| [25] | A2780/cis VS A2780 | fresh cell line | 125b-prec | **199a-prec/335/** **218-prec**/**let-7e**/ ***30c***/ ***126*** /***130a-prec***/ 106a/ 130b/ 26a-prec/23a-prec/ 29c-prec/ 93-prec/ 106-prec/107-prec/ 204-prec/ |

Bold black: Consistent with other experimental results.

Bold Italic with underline: Contrary to other experimental results
